# 1-(4-Chloro­benzo­yl)-2,7-dimethoxy­naphthalene

**DOI:** 10.1107/S1600536808017297

**Published:** 2008-06-19

**Authors:** Ryosuke Mitsui, Kosuke Nakaema, Keiichi Noguchi, Akiko Okamoto, Noriyuki Yonezawa

**Affiliations:** a Department of Organic and Polymer Materials Chemistry, Tokyo University of Agriculture & Technology, 2-24-16 Naka-machi, Koganei, Tokyo 184-8588, Japan; b Instrumentation Analysis Center, Tokyo University of Agriculture & Technology, 2-24-16 Naka-machi, Koganei, Tokyo 184-8588, Japan

## Abstract

In the title compound, C_19_H_15_ClO_3_, the dihedral angle between the naphthalene ring system and the benzene ring is 72.06 (7)°. The 4-chloro­phenyl group and the carbonyl group are almost coplanar. An inter­molecular C—H⋯O hydrogen bond is formed between an H atom of the 4-chloro­phenyl group and the O atom of one meth­oxy group, forming a zigzag chain along the *a* axis.

## Related literature

For the structures of closely related compounds, see: Nakaema *et al.* (2007 ▶); Nakaema, Okamoto *et al.* (2008 ▶); Nakaema, Watanabe *et al.* (2008 ▶).
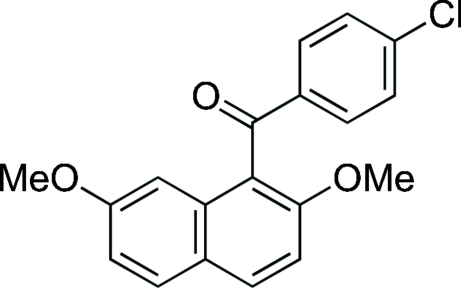


## Experimental

### 

#### Crystal data

C_19_H_15_ClO_3_
*M* *_r_* = 326.76
Orthorhombic, 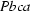

*a* = 6.6033 (3) Å
*b* = 16.0751 (7) Å
*c* = 30.2216 (12) Å
*V* = 3208.0 (2) Å^3^
*Z* = 8
Cu *K*α radiation
μ = 2.21 mm^−1^
*T* = 296 K
0.40 × 0.15 × 0.10 mm

#### Data collection

Rigaku R-AXIS RAPID diffractometer
Absorption correction: multi-scan (**ABSCOR**; Higashi, 1995 ▶) *T*_min_ = 0.617, *T*_max_ = 0.801
54984 measured reflections
2919 independent reflections
2453 reflections with *I* > 2σ(*I*)
*R*_int_ = 0.032

#### Refinement

*R*[*F* ^2^ > 2σ(*F* ^2^)] = 0.040
*wR*(*F* ^2^) = 0.118
*S* = 1.11
2919 reflections
210 parameters
H-atom parameters constrained
Δρ_max_ = 0.13 e Å^−3^
Δρ_min_ = −0.33 e Å^−3^

### 

Data collection: *PROCESS-AUTO* (Rigaku, 1998 ▶); cell refinement: *PROCESS-AUTO*; data reduction: *CrystalStructure* (Rigaku/MSC, 2004 ▶); program(s) used to solve structure: *SIR2004* (Burla *et al.*, 2005 ▶); program(s) used to refine structure: *SHELXL97* (Sheldrick, 2008 ▶); molecular graphics: *ORTEPIII* (Burnett & Johnson, 1996 ▶); software used to prepare material for publication: *SHELXL97*.

## Supplementary Material

Crystal structure: contains datablocks I, global. DOI: 10.1107/S1600536808017297/is2299sup1.cif

Structure factors: contains datablocks I. DOI: 10.1107/S1600536808017297/is2299Isup2.hkl

Additional supplementary materials:  crystallographic information; 3D view; checkCIF report

## Figures and Tables

**Table 1:** Hydrogen-bond geometry (Å, °)

*D*—H⋯*A*	*D*—H	H⋯*A*	*D*⋯*A*	*D*—H⋯*A*
C13—H13⋯O3^i^	0.93	2.58	3.401 (2)	148

Symmetry code: (i) 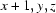.

**Fig. 1.**
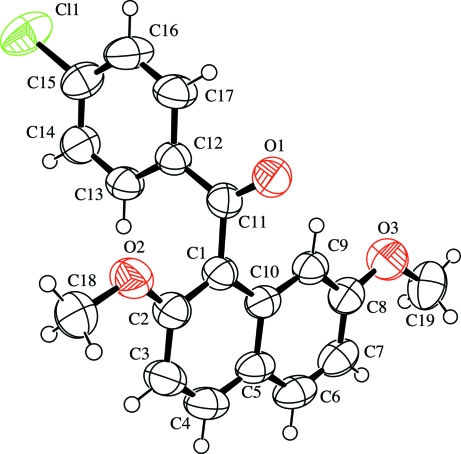
The molecular structure of (I), showing the atom-labeling scheme and 50% probability displacement ellipsoids.

**Fig. 2.**
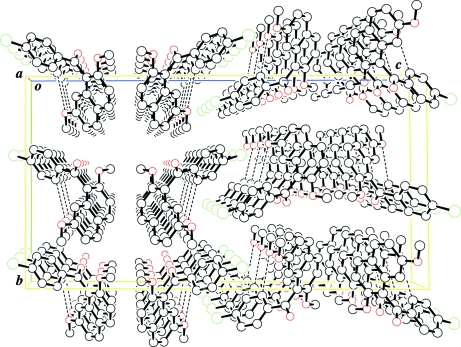
The alignment of the molecules in the crystal structure, viewed along the a axis. H atoms are omitted.

**Fig. 3.**
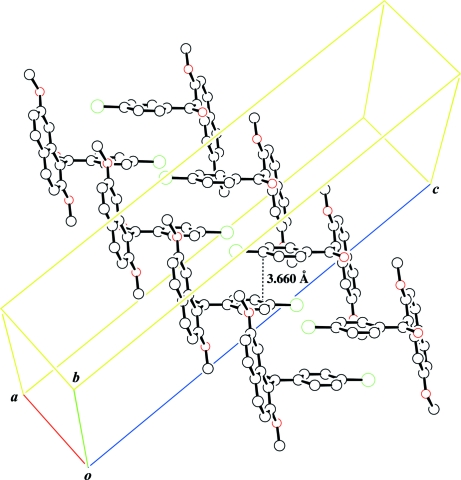
The alignment of the molecules in the crystal structure, viewed in an oblique direction. H atoms are omitted.

**Fig. 4.**
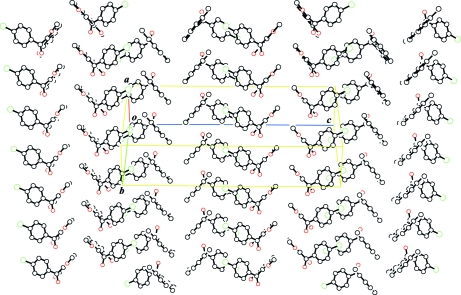
The alignment of the molecules in the crystal structure, showing the herring-bone packing. H atoms are omitted.

## supplementary crystallographic information

### Comment

Recently we have reported the structure of 1,8-bis(4-chlorobenzoyl)-2,7-dimethoxynaphthalene (Nakaema *et al.*, 2007), 2-(4-chlorobenzoyl)-3,6-dimethoxynaphthalene (Nakaema, Okamoto *et al.*, 2008) and 1,8-dibenzoyl-2,7-dimethoxynaphthalene (Nakaema, Watanabe *et al.*, 2008). As part of our ongoing studies on the formation reaction and structure of the aroylated naphthalene derivatives synthesis and crystal structure analysis of the title compound, (I), were performed. The title compound was prepared by electrophilic aromatic aroylation reaction of 2,7-dimethoxynaphthalene with 4-chlorobenzoyl chloride.

An *ORTEPIII* (Burnett & Johnson, 1996) plot of (I) is displayed in Fig. 1. In the molecule of (I), the interplanar angle between the benzene ring (C12—C17) and the naphthalene ring (C1—C10) is 72.06 (7)°. The carbonyl group and the 4-chlorophenyl group are almost coplanar [O1—C11—C12—C17 torsion angle = -4.4 (2)°].

In the crystal structure, the molecular packing of (I) is mainly stabilized by van der Waals interaction. The molecules of (I) are aligned consecutively in stacks along the *a* axis (Fig. 2). Adjacent 4-chlorophenyl groups are exactly parallel, and the perpendicular distance between these planes is 3.660 (1) Å (Fig. 3). Figure 4 shows the herring-bone packing of the naphthalene ring in the crystal. The crystal packing is additionally stabilized by intermolecular C—H···O hydrogen bonding between the methoxy oxygen and a hydrogen atom of the nearby 4-chlorophenyl group of the adjacent molecule (C13—H13···O3^i^; Fig. 2 and Table 1).

### Experimental

To a solution of 4-chlorobenzoyl chloride (77 mg, 0.44 mmol) and AlCl_3_ (64 mg, 0.48 mmol) in nitrobenzene (1.0 ml) was added a solution of 2,7-dimethoxynaphthalene (0.40 *M* in nitrobenzene, 1.0 ml, 0.40 mmol) drop-wise at 0 °C. The reaction mixture was stirred for 6 h at 0 °C and immediately poured into H_2_O (10 ml) and CHCl_3_ (5 ml). The aqueous layer was extracted with CHCl_3_ (3 × 5 ml). The combined organic layers were washed with aqueous 2 *M* NaOH (3 × 20 ml), brine (3 × 20 ml), and dried over MgSO_4_ for overnight. The solvent was removed *in vacuo* and the crude material was purified by recrystallization from hexanes to give the title compound as a colorless platelets (m.p. 394.5–394.8 K, yield 102 mg, 78%).

Spectroscopic Data: ^1^H NMR (300 MHz, CDCl_3_) δ 7.87 (d, 1H), 7.78 (d, 2H), 7.72 (d, 1H), 7.39 (d, 2H), 7.15 (d, 1H), 7.02 (dd, 1H), 6.78 (d, 1H), 3.79 (s, 3H), 3.73 (s, 3H); ^13^C NMR (75 MHz, CDCl_3_) δ 196.7, 159.0, 155.0, 139.7, 136.5, 133.0, 131.3, 130.8, 129.7, 128.8, 124.4, 121.1, 117.1, 110.1, 102.0, 56.2, 55.2; IR (KBr): 1667, 1628, 1587, 1575, 1513, 1278, 1241, 1047.

Anal. Calcd for C_19_H_15_ClO_3_: C 69.84, H 4.63. Found: C 69.61, H 4.74.

### Refinement

All H atoms were found in a difference map and were subsequently refined as riding atoms, with C—H = 0.93 (aromatic) and 0.96 (methyl) Å, and with *U*_iso_(H) = 1.2*U*_eq_(C).

### Crystal data

**Table:**

C_19_H_15_ClO_3_	*D*_x_ = 1.353 Mg m^−^^3^
*M**_r_* = 326.76	Melting point = 394.5–394.8 K
Orthorhombic, *P**b**c**a*	Cu *K*α radiation λ = 1.54187 Å
Hall symbol: -P 2ac 2ab	Cell parameters from 46869 reflections
*a* = 6.6033 (3) Å	θ = 3.1–68.1º
*b* = 16.0751 (7) Å	µ = 2.21 mm^−^^1^
*c* = 30.2216 (12) Å	*T* = 296 K
*V* = 3208.0 (2) Å^3^	Platelet, colorless
*Z* = 8	0.40 × 0.15 × 0.10 mm
*F*_000_ = 1360	

### Data collection

**Table:**

Rigaku R-AXIS RAPID diffractometer	2919 independent reflections
Radiation source: rotating anode	2453 reflections with *I* > 2σ(*I*)
Monochromator: graphite	*R*_int_ = 0.032
Detector resolution: 10.00 pixels mm^-1^	θ_max_ = 68.1º
*T* = 296 K	θ_min_ = 5.5º
ω scans	*h* = −7→7
Absorption correction: multi-scan(*ABSCOR*; Higashi, 1995)	*k* = −19→19
*T*_min_ = 0.617, *T*_max_ = 0.801	*l* = −36→36
54984 measured reflections	

### Refinement

**Table:**

Refinement on *F*^2^	Secondary atom site location: difference Fourier map
Least-squares matrix: full	Hydrogen site location: difference Fourier map
*R*[*F*^2^ > 2σ(*F*^2^)] = 0.040	H-atom parameters constrained
*wR*(*F*^2^) = 0.118	*w* = 1/[σ^2^(*F*_o_^2^) + (0.057*P*)^2^ + 0.6411*P*] where *P* = (*F*_o_^2^ + 2*F*_c_^2^)/3
*S* = 1.11	(Δ/σ)_max_ < 0.001
2919 reflections	Δρ_max_ = 0.13 e Å^−^^3^
210 parameters	Δρ_min_ = −0.33 e Å^−^^3^
Primary atom site location: structure-invariant direct methods	Extinction correction: none

### Special details

**Table:**

Geometry. All e.s.d.'s (except the e.s.d. in the dihedral angle between two l.s. planes) are estimated using the full covariance matrix. The cell e.s.d.'s are taken into account individually in the estimation of e.s.d.'s in distances, angles and torsion angles; correlations between e.s.d.'s in cell parameters are only used when they are defined by crystal symmetry. An approximate (isotropic) treatment of cell e.s.d.'s is used for estimating e.s.d.'s involving l.s. planes.
Refinement. Refinement of *F*^2^ against ALL reflections. The weighted *R*-factor *wR* and goodness of fit *S* are based on *F*^2^, conventional *R*-factors *R* are based on *F*, with *F* set to zero for negative *F*^2^. The threshold expression of *F*^2^ > σ(*F*^2^) is used only for calculating *R*-factors(gt) *etc*. and is not relevant to the choice of reflections for refinement. *R*-factors based on *F*^2^ are statistically about twice as large as those based on *F*, and *R*- factors based on ALL data will be even larger.

### Fractional atomic coordinates and isotropic or equivalent isotropic displacement parameters (Å^2^)

**Table:**

	*x*	*y*	*z*	*U*_iso_*/*U*_eq_	
Cl1	1.30817 (12)	−0.12548 (4)	−0.02595 (2)	0.1046 (3)	
O1	0.6267 (2)	−0.08777 (8)	0.13581 (5)	0.0831 (4)	
O2	1.0971 (2)	−0.05318 (8)	0.18610 (5)	0.0795 (4)	
O3	0.2593 (2)	0.17844 (8)	0.10247 (5)	0.0867 (4)	
C1	0.8299 (2)	0.02580 (10)	0.15836 (5)	0.0558 (4)	
C2	0.9962 (3)	0.02106 (11)	0.18585 (6)	0.0631 (4)	
C3	1.0529 (3)	0.08948 (13)	0.21247 (6)	0.0725 (5)	
H3	1.1655	0.0861	0.2308	0.087*	
C4	0.9409 (3)	0.16017 (12)	0.21089 (6)	0.0730 (5)	
H4	0.9805	0.2053	0.2281	0.088*	
C5	0.7672 (3)	0.16779 (10)	0.18420 (5)	0.0616 (4)	
C6	0.6488 (3)	0.24079 (11)	0.18207 (6)	0.0725 (5)	
H6	0.6863	0.2864	0.1991	0.087*	
C7	0.4820 (3)	0.24673 (11)	0.15594 (6)	0.0723 (5)	
H7	0.4072	0.2957	0.1552	0.087*	
C8	0.4234 (3)	0.17821 (10)	0.13003 (6)	0.0644 (4)	
C9	0.5338 (3)	0.10614 (10)	0.13091 (5)	0.0588 (4)	
H9	0.4928	0.0612	0.1137	0.071*	
C10	0.7086 (2)	0.09875 (10)	0.15751 (5)	0.0546 (4)	
C11	0.7780 (2)	−0.04641 (10)	0.12873 (6)	0.0572 (4)	
C12	0.9101 (2)	−0.06394 (9)	0.09012 (5)	0.0542 (4)	
C13	1.0749 (3)	−0.01439 (10)	0.07974 (6)	0.0632 (4)	
H13	1.1037	0.0319	0.0971	0.076*	
C14	1.1968 (3)	−0.03274 (12)	0.04398 (6)	0.0718 (5)	
H14	1.3074	0.0007	0.0372	0.086*	
C15	1.1529 (3)	−0.10086 (11)	0.01855 (6)	0.0700 (5)	
C16	0.9900 (4)	−0.15030 (13)	0.02771 (7)	0.0839 (6)	
H16	0.9615	−0.1961	0.0100	0.101*	
C17	0.8687 (3)	−0.13182 (11)	0.06334 (7)	0.0736 (5)	
H17	0.7573	−0.1653	0.0695	0.088*	
C18	1.2729 (3)	−0.06178 (17)	0.21273 (7)	0.0894 (6)	
H18A	1.3333	−0.1152	0.2075	0.107*	
H18B	1.2363	−0.0571	0.2434	0.107*	
H18C	1.3681	−0.0188	0.2053	0.107*	
C19	0.1263 (3)	0.24781 (13)	0.10308 (9)	0.0925 (7)	
H19A	0.0139	0.2374	0.0837	0.111*	
H19B	0.1976	0.2965	0.0933	0.111*	
H19C	0.0775	0.2564	0.1326	0.111*	

### Atomic displacement parameters (Å^2^)

**Table:**

	*U*^11^	*U*^22^	*U*^33^	*U*^12^	*U*^13^	*U*^23^
Cl1	0.1391 (6)	0.0859 (4)	0.0887 (4)	0.0050 (3)	0.0444 (4)	−0.0088 (3)
O1	0.0689 (8)	0.0647 (8)	0.1156 (11)	−0.0185 (6)	0.0228 (7)	−0.0237 (7)
O2	0.0751 (8)	0.0765 (9)	0.0869 (9)	0.0094 (7)	−0.0210 (7)	−0.0126 (7)
O3	0.0830 (9)	0.0638 (8)	0.1132 (11)	0.0160 (7)	−0.0117 (8)	−0.0033 (7)
C1	0.0563 (9)	0.0530 (8)	0.0580 (9)	−0.0081 (7)	0.0046 (7)	−0.0055 (7)
C2	0.0620 (10)	0.0644 (10)	0.0630 (10)	−0.0064 (8)	0.0019 (8)	−0.0055 (7)
C3	0.0746 (12)	0.0808 (13)	0.0622 (10)	−0.0154 (10)	−0.0051 (9)	−0.0111 (9)
C4	0.0908 (14)	0.0668 (11)	0.0613 (10)	−0.0239 (10)	0.0043 (9)	−0.0148 (8)
C5	0.0772 (11)	0.0532 (9)	0.0544 (8)	−0.0148 (8)	0.0137 (8)	−0.0073 (7)
C6	0.1007 (14)	0.0491 (9)	0.0677 (11)	−0.0127 (9)	0.0175 (10)	−0.0101 (7)
C7	0.0923 (13)	0.0464 (8)	0.0781 (12)	0.0022 (9)	0.0210 (11)	−0.0005 (8)
C8	0.0694 (11)	0.0526 (9)	0.0711 (10)	−0.0012 (8)	0.0096 (9)	0.0029 (7)
C9	0.0645 (10)	0.0483 (8)	0.0636 (9)	−0.0046 (7)	0.0062 (8)	−0.0052 (7)
C10	0.0628 (9)	0.0472 (8)	0.0539 (8)	−0.0094 (7)	0.0117 (7)	−0.0028 (6)
C11	0.0539 (9)	0.0465 (8)	0.0711 (10)	−0.0030 (7)	−0.0005 (7)	−0.0041 (7)
C12	0.0574 (9)	0.0442 (7)	0.0611 (9)	−0.0014 (7)	−0.0045 (7)	−0.0021 (6)
C13	0.0671 (10)	0.0543 (9)	0.0681 (10)	−0.0097 (8)	0.0017 (8)	−0.0088 (7)
C14	0.0748 (12)	0.0653 (11)	0.0752 (11)	−0.0092 (9)	0.0109 (9)	−0.0003 (9)
C15	0.0908 (13)	0.0565 (9)	0.0628 (10)	0.0047 (9)	0.0110 (9)	0.0016 (8)
C16	0.1138 (16)	0.0636 (11)	0.0742 (12)	−0.0172 (12)	0.0141 (11)	−0.0208 (9)
C17	0.0850 (12)	0.0592 (10)	0.0766 (12)	−0.0206 (9)	0.0066 (10)	−0.0143 (8)
C18	0.0724 (13)	0.1075 (17)	0.0884 (14)	0.0130 (12)	−0.0139 (11)	−0.0099 (12)
C19	0.0852 (14)	0.0726 (13)	0.1198 (18)	0.0214 (11)	0.0122 (13)	0.0190 (12)

### Geometric parameters (Å, °)

**Table:**

Cl1—C15	1.7366 (19)	C8—C9	1.369 (2)
O1—C11	1.219 (2)	C9—C10	1.412 (2)
O2—C2	1.367 (2)	C9—H9	0.9300
O2—C18	1.419 (2)	C11—C12	1.484 (2)
O3—C8	1.367 (2)	C12—C13	1.384 (2)
O3—C19	1.420 (2)	C12—C17	1.386 (2)
C1—C2	1.379 (2)	C13—C14	1.380 (2)
C1—C10	1.420 (2)	C13—H13	0.9300
C1—C11	1.506 (2)	C14—C15	1.369 (3)
C2—C3	1.413 (2)	C14—H14	0.9300
C3—C4	1.356 (3)	C15—C16	1.366 (3)
C3—H3	0.9300	C16—C17	1.375 (3)
C4—C5	1.408 (3)	C16—H16	0.9300
C4—H4	0.9300	C17—H17	0.9300
C5—C6	1.411 (3)	C18—H18A	0.9600
C5—C10	1.426 (2)	C18—H18B	0.9600
C6—C7	1.359 (3)	C18—H18C	0.9600
C6—H6	0.9300	C19—H19A	0.9600
C7—C8	1.406 (3)	C19—H19B	0.9600
C7—H7	0.9300	C19—H19C	0.9600
			
C2—O2—C18	119.13 (16)	O1—C11—C1	120.20 (15)
C8—O3—C19	119.00 (17)	C12—C11—C1	118.69 (13)
C2—C1—C10	120.37 (15)	C13—C12—C17	118.41 (16)
C2—C1—C11	119.81 (15)	C13—C12—C11	122.07 (14)
C10—C1—C11	119.82 (14)	C17—C12—C11	119.52 (15)
O2—C2—C1	116.08 (14)	C14—C13—C12	120.88 (16)
O2—C2—C3	123.20 (17)	C14—C13—H13	119.6
C1—C2—C3	120.71 (17)	C12—C13—H13	119.6
C4—C3—C2	119.21 (18)	C15—C14—C13	119.16 (17)
C4—C3—H3	120.4	C15—C14—H14	120.4
C2—C3—H3	120.4	C13—C14—H14	120.4
C3—C4—C5	122.49 (16)	C16—C15—C14	121.23 (18)
C3—C4—H4	118.8	C16—C15—Cl1	119.29 (15)
C5—C4—H4	118.8	C14—C15—Cl1	119.48 (15)
C4—C5—C6	123.35 (16)	C15—C16—C17	119.48 (17)
C4—C5—C10	118.52 (17)	C15—C16—H16	120.3
C6—C5—C10	118.12 (17)	C17—C16—H16	120.3
C7—C6—C5	122.26 (16)	C16—C17—C12	120.83 (18)
C7—C6—H6	118.9	C16—C17—H17	119.6
C5—C6—H6	118.9	C12—C17—H17	119.6
C6—C7—C8	119.47 (17)	O2—C18—H18A	109.5
C6—C7—H7	120.3	O2—C18—H18B	109.5
C8—C7—H7	120.3	H18A—C18—H18B	109.5
O3—C8—C9	115.86 (15)	O2—C18—H18C	109.5
O3—C8—C7	123.76 (16)	H18A—C18—H18C	109.5
C9—C8—C7	120.37 (18)	H18B—C18—H18C	109.5
C8—C9—C10	121.17 (15)	O3—C19—H19A	109.5
C8—C9—H9	119.4	O3—C19—H19B	109.5
C10—C9—H9	119.4	H19A—C19—H19B	109.5
C9—C10—C1	122.75 (14)	O3—C19—H19C	109.5
C9—C10—C5	118.59 (15)	H19A—C19—H19C	109.5
C1—C10—C5	118.66 (15)	H19B—C19—H19C	109.5
O1—C11—C12	121.07 (15)		
			
C18—O2—C2—C1	178.33 (18)	C2—C1—C10—C5	2.5 (2)
C18—O2—C2—C3	−2.9 (3)	C11—C1—C10—C5	−176.81 (14)
C10—C1—C2—O2	176.63 (15)	C4—C5—C10—C9	179.66 (15)
C11—C1—C2—O2	−4.0 (2)	C6—C5—C10—C9	−1.1 (2)
C10—C1—C2—C3	−2.2 (2)	C4—C5—C10—C1	−1.1 (2)
C11—C1—C2—C3	177.18 (16)	C6—C5—C10—C1	178.16 (14)
O2—C2—C3—C4	−178.39 (17)	C2—C1—C11—O1	110.8 (2)
C1—C2—C3—C4	0.3 (3)	C10—C1—C11—O1	−69.8 (2)
C2—C3—C4—C5	1.1 (3)	C2—C1—C11—C12	−71.3 (2)
C3—C4—C5—C6	−179.95 (17)	C10—C1—C11—C12	108.01 (17)
C3—C4—C5—C10	−0.7 (3)	O1—C11—C12—C13	175.57 (17)
C4—C5—C6—C7	179.79 (17)	C1—C11—C12—C13	−2.2 (2)
C10—C5—C6—C7	0.6 (3)	O1—C11—C12—C17	−4.4 (3)
C5—C6—C7—C8	0.1 (3)	C1—C11—C12—C17	177.83 (16)
C19—O3—C8—C9	173.79 (17)	C17—C12—C13—C14	−0.9 (3)
C19—O3—C8—C7	−7.0 (3)	C11—C12—C13—C14	179.13 (17)
C6—C7—C8—O3	−179.41 (17)	C12—C13—C14—C15	0.2 (3)
C6—C7—C8—C9	−0.3 (3)	C13—C14—C15—C16	0.6 (3)
O3—C8—C9—C10	178.92 (15)	C13—C14—C15—Cl1	−178.88 (15)
C7—C8—C9—C10	−0.3 (3)	C14—C15—C16—C17	−0.5 (3)
C8—C9—C10—C1	−178.25 (15)	Cl1—C15—C16—C17	178.95 (18)
C8—C9—C10—C5	1.0 (2)	C15—C16—C17—C12	−0.3 (3)
C2—C1—C10—C9	−178.27 (15)	C13—C12—C17—C16	1.0 (3)
C11—C1—C10—C9	2.4 (2)	C11—C12—C17—C16	−179.07 (19)

### Hydrogen-bond geometry (Å, °)

**Table:**

*D*—H···*A*	*D*—H	H···*A*	*D*···*A*	*D*—H···*A*
C13—H13···O3^i^	0.93	2.58	3.401 (2)	148

Symmetry codes: (i) *x*+1, *y*, *z*.
